# Conditional *Dnmt3b* deletion in hippocampal dCA1 impairs recognition memory

**DOI:** 10.1186/s13041-020-00574-9

**Published:** 2020-03-17

**Authors:** Qingnuan Kong, Ming Yu, Meng Zhang, Chuang Wei, Huating Gu, Shaoyang Yu, Wei Sun, Nan Li, Yu Zhou

**Affiliations:** 1 Department of Physiology and Pathophysiology, School of Basic Medical Sciences, Qingdao University, Qingdao, 266071 Shandong China; 2 Department of Pathology, Qingdao Municipal hospital, Qingdao University, Qingdao, 266071 Shandong China; 3 Institute of Brain Sciences and Related Disorders, Qingdao University, Qingdao, 266071 Shandong China

**Keywords:** *Dnmt3b*, Memory, Hippocampus, Object-place recognition

## Abstract

**Aim:**

Active changes in neuronal DNA methylation and demethylation appear to act as controllers of synaptic scaling and glutamate receptor trafficking in learning and memory formation. DNA methyltransferases (DNMTs), including proteins encoded by *Dnmt1*, *Dnmt3a* and *Dnmt3b*, are dominant enzymes carrying out DNA methylation. Our previous study demonstrated the important roles that DNMT1 and DNMT3a play in synaptic function and memory. In this study, we aim to explore the role of DNMT3b and its-mediated DNA methylation in memory processes.

**Methods:**

*Dnmt3b* was knocked down specifically in dorsal CA1 neurons of adult mice hippocampus by AAV-syn-Cre-GFP virus injection. Behavioral tests were used to evaluate memory performance. Gene expression microarray analysis followed by quantitative RT-PCR were performed to find differential expression genes.

**Results:**

*Dnmt3b*^*flox/flox*^ mice receiving Cre-virus infection showed impaired novel object-place recognition (NPR) and normal novel object recognition (NOR), in comparison to mice receiving control GFP-virus infection. Microarray analysis revealed differential expression of K^+^ channel subunits in the hippocampus of *Dnmt3b*^*flox/flox*^ mice receiving Cre-virus injection. Increased *Kcne2* expression was confirmed by following qRT-PCR analysis. We also found that NPR training and testing induced up-regulation of hippocampal *Dnmt1* and *Dnmt3a* mRNA expression in control mice, but not in Cre-virus injected mice. Our findings thus demonstrate that conditional *Dnmt3b deletion* in a sub-region of the hippocampus impairs a specific form of recognition memory that is hippocampus-dependent.

## Main text

It is well-known that DNA methylation alters gene expression without changing DNA sequence, and it plays a vital role in regulating adult brain functions including learning and memory [1, 2]. Accumulative evidence has revealed that DNA methylation alterations in brain neurons dynamically modulates synaptic plasticity and are required for multiple forms of memory formation, for example contextual fear memory, object recognition memory, and spatial memory [1–3]. DNA methylation is catalyzed by DNA methyltransferases (DNMTs), which add methyl to the 5′ position of cytosine (C) to form a 5-methyl cytosine (5mC). DNMT1, DNMT3a and DNMT3b are three active DNMTs identified in mammals. DNMT3L enhances the DNA methylation activity of other DNMT3 whereas it is catalytically inactive. It is noted that DNMTs in neurons are closely associated with learning and memory processes [4, 5]. Our previous findings showed that double knockout of *Dnmt1* and *Dnmt3a* in αCaMKII^+^ forebrain neurons led to hippocampus-dependent memory impairment [1]. In spite of very limited expression in mature neurons, several studies have suggested that DNMT3b, when concurrently functioning with DNMT1 or DNMT3a to modulate DNA methylation, may also play an important role in controlling gene expression and memory processes [6–8]. Besides, DNMT3b alone has been proven to play specific roles in regulating methylation and certain brain functions. Suicide attempters were linked to *Dnmt3b* SNP polymorphisms [9]. EPO micro-injected into the hippocampus upregulated *Dnmt3b* expression and improved spatial learning & memory in SAMP8 mice [10]. However, its molecular and cellular mechanisms remain unclear. In the present study, we conducted Cre-dependent neuronal *Dnmt3b* deletion specifically in CA1 region (dCA1) of the dorsal hippocampus in order to explore memory alterations and underlying molecular mechanisms.

**Fig. 1:**
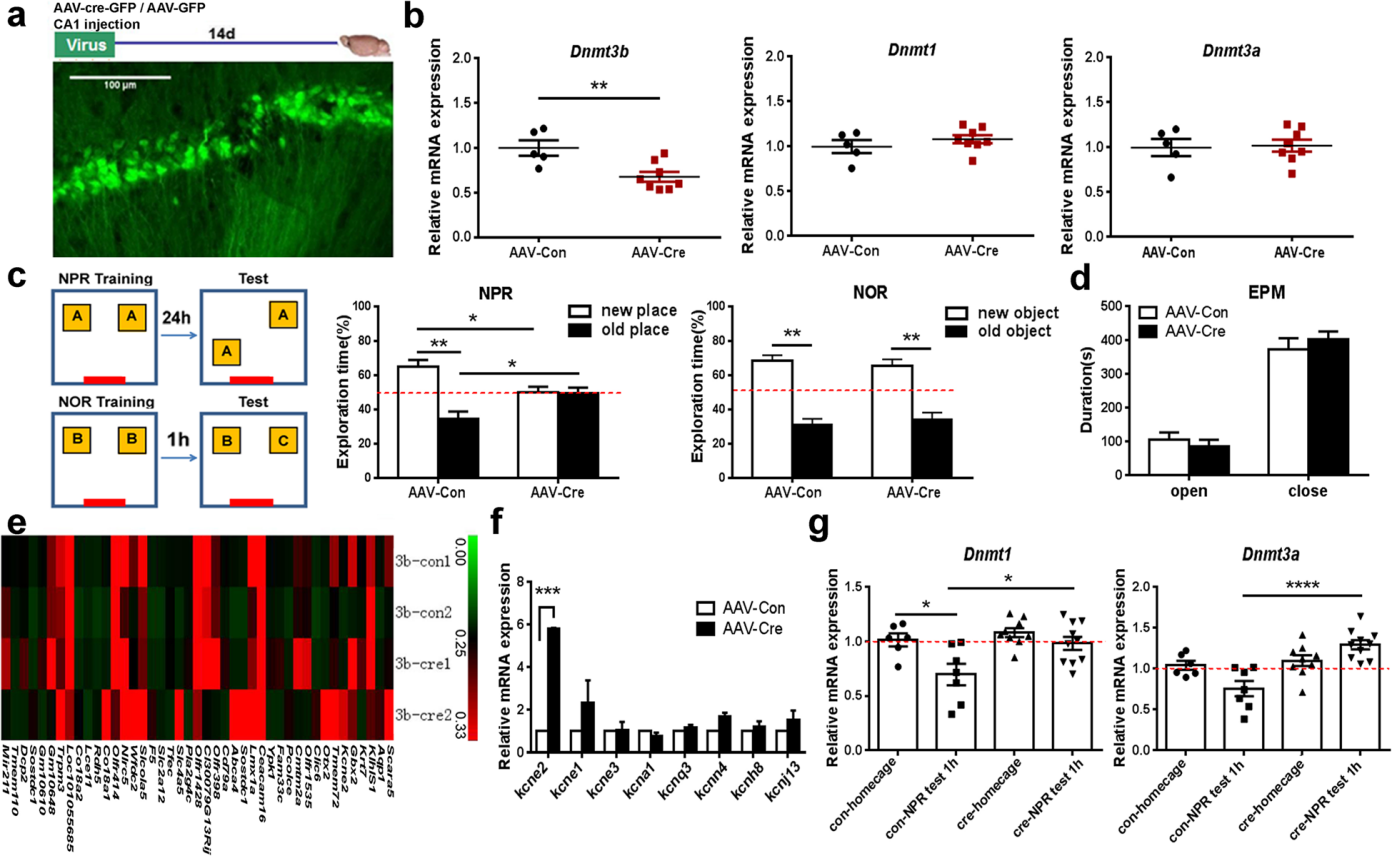
Conditional *Dnmt3b* deletion in CA1 neurons of dorsal hippocampus impairs object-place recognition memory. **a** Representative image showing virus infection (GFP expression) in dCA1 neurons at 14d after injection. **b** Quantitative RT-PCR showing relative *Dnmt3b* (left), *Dnmt1* (middle) and *Dnmt3a* (right) mRNA expression in the hippocampus. Unpaired *t* test, *n* = 5 for control-virus group and *n* = 8 for Cre-virus group. **c** Cre-expressing *Dnmt3b*^*flox/flox*^ mice showing NPR memory deficits with normal NOR memory. **Left,** Illustration of NPR and NOR behavior paradigms. **Middle,** NPR test. **Right**, NOR test. Two-way ANOVA followed by Sidak’s multiple comparisons test, n = 5 for control-virus group and n = 8 for Cre-virus group. **d***Dnmt3b* deletion in CA1 of dorsal hippocampus did not affect anxiety-like behavior. Two-way ANOVA followed by Sidak’s multiple comparisons test, *n* = 11 for control group and *n* = 18 for Cre-virus group. **e** The heatmap of differentially expressed genes. **f** Quantitative RT-PCR analysis showing increased *Kcne2* expression after *Dnmt3b* deletion. Two-way ANOVA followed by Bonferroni posttests. **g***Dnmt3b* deletion blocked dynamic changes of *Dnmt1* and *Dnmt3a* mRNA expression induced by NPR training and test. **Left,***Dnmt1* expression. **Right**, *Dnmt3a* expression. One-way ANOVA followed by Tukey’s multiple comparisons test, *n* = 6 for control group at home cage, *n* = 7 for control group at 1 h after NPR test, *n* = 9 for Cre-expressing group at home cage, *n* = 10 for Cre-expressing group at 1 h after NPR test. ^∗^*P* < 0.05, ^∗∗^*P* < 0.01, and ^∗∗∗^*P* < 0.001 indicates significant difference between compared groups. All data are present as means ± SEM

Cre- or Con- virus was delivered to hippocampal dCA1 of *Dnmt3b*^*flox/flox*^ mice by microinjection. Gene expression microarray analysis and a following quantitative RT-PCR analysis were performed to explore the differential expression of candidate genes. The detailed methods were described in the Additional file 1. GFP fluorescence indicated that dCA1 neurons in the hippocampus were successfully transfected at 14 days after the local virus injection (Fig. 1a). Quantitative RT-PCR analysis showed that *Dnmt3b* mRNA expression in the hippocampus was reduced in *Dnmt3b*^*flox/flox*^ mice receiving the Cre-virus injection, while the *Dnmt1* and *Dnmt3a* mRNA expressions were similar between two groups of mice (Fig. 1b). Then we tested whether Cre-dependent *Dnmt3b* deletion in adult hippocampus of *Dnmt3b*^*flox/flox*^ mice affects learning and memory. *Dnmt3b*^*flox/flox*^ mice receiving Cre-virus injection exhibited object-place recognition deficits but normal object recognition memory (Fig. 1c). In addition, *Dnmt3b*^*flox/flox*^ mice receiving Cre-virus injection exhibited normal spatial learning and memory in a Morris water maze task (data not shown). Also, an elevated plus maze test disclosed that Cre-dependent *Dnmt3b* deletion in CA1 neurons of dorsal hippocampus did not affect anxiety-like behavior of *Dnmt3b*^*flox/flox*^ mice (Fig. 1d). It was reported that object-place memory and object memory appear to be dependent on different brain regions. Object-place memory requires the hippocampus for encoding, consolidation and retrieval [11, 12], and it is particularly sensitive to manipulations in dorsal CA1 [13]; whereas object memory requires the participation of different brain regions including insular cortex, perirhineal cortex and medial prefrontal cortex [14]. The role of the hippocampus in object recognition has remained controversial [11]. Therefore, with our virus-based, Cre-dependent *Dnmt3b* deletion system, it might be interesting to test whether *Dnmt3b* deletion in other brain regions, such as insular cortex or perirhineal cortex, affects object recognition rather than object-place recognition.

To explore the molecular mechanism underlying object-place recognition memory deficits caused by Cre-dependent *Dnmt3b* deletion in dorsal hippocampal neurons, we extracted total RNA from dorsal hippocampus of *Dnmt3b*^*flox/flox*^ mice receiving either Cre- or control- virus injection. Gene expression microarray analysis showed forty-six differentially expressed genes with folder changes over 1.5 times, including 22 upregulated genes and 24 downregulated genes (Fig. 1e). Among those 46 genes, *Kcne2* expression was significantly upregulated, which was then confirmed by real-time qRT-PCR analysis (Fig. 1f). It is reported that KCNE2 modulates neuronal excitability through regulating Kv channel activity in the brain [15], although so far there is no direct evidence proving that KCNE family is involved in neuron plasticity and memory. Therefore, we presumed that the upregulation of *Kcne2* expression caused by *Dnmt3b* deletion in dorsal hippocampus might contribute to the NPR deficits observed in *Dnmt3b*^*flox/flox*^ mice.

Moreover, we found that, object-place recognition learning and memory process was accompanied by dynamic changes of hippocampal *Dnmt1* and *Dnmt3a* mRNA expression in *Dnmt3b*^*flox/flox*^ mice receiving control-virus injection, while not in *Dnmt3b*^*flox/flox*^ mice receiving Cre-virus injection (Fig. 1g). *Dnmt1* mRNA expression in the hippocampus of control *Dnmt3b*^*flox/flox*^ mice significantly decreased after NPR training and testing (Fig. 1g). However, NPR training and testing did not change the *Dnmt1* mRNA level in *Dnmt3b*^*flox/flox*^ mice receiving Cre-virus injection (Fig. 1g). After NPR training and testing, hippocampal *Dnmt1* mRNA expression showed significant difference between *Dnmt3b*^*flox/flox*^ mice receiving Cre-virus and *Dnmt3b*^*flox/flox*^ mice receiving control-virus injection (Fig. 1g). Similar to dynamic change of *Dnmt1*, *Dnmt3a* mRNA expression also slightly decreased following NPR training and testing in control *Dnmt3b*^*flox/flox*^ mice, but not in Cre-expressing *Dnmt3b*^*flox/flox*^ mice. Significantly, *Dnmt3b*^*flox/flox*^ mice infected by Cre-virus displayed even higher hippocampal *Dnmt3a* expression than *Dnmt3b*^*flox/flox*^ mice infected by control-virus (Fig. 1g). Therefore, our results demonstrated that Cre-dependent *Dnmt3b* deletion blocked dynamic down-regulation of *Dnmt1* and *Dnmt3a* mRNA expression induced by NPR training and testing, which may also contribute to NPR deficits observed in those *Dnmt3b*^*flox/flox*^ mice.

In conclusion, our study suggests that DNMT3b in mature neurons of dorsal hippocampus especially at the CA1 region plays an important role in regulating object-place recognition memory. We postulate that *Kcne2* is one of the important genes targeted by DNMT3b*-*mediated DNA methylation, therefore contributes to object-place recognition process.

## Supplementary information

**Additional file 1.**

## Data Availability

The detailed methods are described in the Additional file 1.

## Abbreviations

AAV: Adeno-associated virus
DNMT: DNA methyltransferases
EPO: Erythropoietin
GFP: Green fluorescent protein
*Kcne*: Potassium voltage-gated channel subfamily E member 2
NOR: Novel object recognition
NPR: Novel object-place recognition
PCR: Polymerase chain reaction
SAMP8: Senescence accelerated mouse-prone 8
SNP: Single nucleotide polymorphism
αCaMKII: Ca ^2+^/calmodulin-dependent protein kinase IIα
