# Supplementary material for: Conditional Dnmt3b deletion in hippocampal dCA1 impairs recognition memory
Source: Mol Brain. 2020 Mar 17;13:42. doi: 10.1186/s13041-020-00574-9 (PMC7079487; doi:10.1186/s13041-020-00574-9)
Supplement: Supplementary file 1 — Additional file 1. [file 13041_2020_574_MOESM1_ESM.docx]

**Method:**

**Mice:** Group-housed *Dnmt3b^flox/flox^* male mice at 3- to 6-month old were included in this study. The animals were maintained under standard conditions with 12/12h light/dark circle and 22+/-2℃ temperature. Food and water were freely accessible. The Chancellor’s Animal Research Committee at Qingdao University approved all the experiments according to National Institutes of Health guideline.

**Behavior:**

**Elevated Plus Maze (EPM) test**

EPM test is a behavioral paradigm commonly used to measure rodents’ anxiety-like behavior. The maze is 45cm high and composed of two closed arms (35 cm in length, 6 cm in width, and 25 cm in height) and two open arms (35 cm in length, 6cm in width, and 0.5 cm in height) with a central square (10 x10 cm). Each animal was gently placed at the center area facing to one of the closed arm and allowed to freely explore the maze for 10 min. The distance traveled, the number of entries into each arm and the time spent in each arm are automatically recorded and analyzed by Noldus EthoVision XT video-tracking software.

**Novel Object recognition (NOR) and Novel Object-Place Recognition (NPR) test**

NOR and NPR tests were performed in a square, non-transparent plastic box (28x28x40 cm) with a visual cue hanging on one side of the walls. All mice were handled for 3 days (2 min per day) and habituated in the empty testing box for another 3 days (10 min per day) prior to training. During a NPR or NOR training session, mice were allowed to explore two identical objects placed in the testing box for 10 min. Each animal received three times training with a 30 min intervals. NPR test was done 24 hours after training, during which one object was moved to a novel location and mice were allowed to explore the same testing box for 5 min. NOR memory was tested at 1h after training, during which one object was replaced by a novel object. Close sniffing or touching the objects was considered to be an effective object exploration. Percentage of time exploring the novel or old object-place was compared to evaluate recognition memory. Recognition Memory Index (%) was calculated as novel object exploration time/total exploration time x 100%.

**Virus injections**

Mice were anaesthetized by intraperitoneal injection of 5% Chloral hydrate (0.1mg/10g) and fixed on a stereotaxic frame. AAV-syn-Cre-GFP (AAV-Cre) virus or AAV-syn-GFP control (AAV-Con) virus was bilaterally microinjected into dCA1 areas of the hippocampus according to the following coordinates: AP -1.8mm, ML +/-1.0mm, DV -1.4mm; AP -2.5mm, ML +/-2.0mm, DV -1.7mm. Virus was delivered with a 10 ml Hamilton syringe with 1ul per hole at a 0.05 ul/min rate. To guarantee infection efficiency, the needle was left in brain for an additional 10min after injection to prevent backflow of virus suspension. After surgery, mice were maintained in the same home cage for 14 days. Green GFP fluorescence was used to confirm infection position and Dnmt3b deletion rate was confirmed by real-time qRT-PCR. Behavior procedures were performed 4–6 weeks following virus injection.

**Gene expression Microarray and Quantitative qRT-PCR**

Total RNA was extracted from the hippocampus using PureLink® RNA kit according to the manufacturer’s instructions (Invitrogen). RNA was then quantified with NanoDrop ND-2000 (Thermo Scientific) and the RNA integrity was assessed using Agilent Bioanalyzer 2100 (Agilent Technologies). Reverse transcription was performed using RT^2^ First Strand Kit (Qiagen) or SuperScript III First-Strand Synthesis kit (Invitrogen) according to manufactor’s instrucitons.

Agilent Mouse Gene Expression (8x60K) analysis was done by collaboration with Shanghai OE Biotech Co Ltd (China). Briefly, total RNA were transcribed to double strand cDNA, then synthesized into cRNA and labeled with Cy3. The labeled cRNAs were hybridized onto microarray (Agilent SurePrint G3 Mouse Gene Expression v2.0 Microarray 8x60K). After washing, the arrays were scanned by the Agilent Scanner G2505C (Agilent Technologies). Feature Extraction software (version10.7.1.1, Agilent Technologies) was used to get raw data and Genespring (version 12.5; Agilent Technologies) were then employed to normalize data with quantile algorithm. Genes with differential expression were identified through fold change as well as P value calculated with student’s unpaired t-test. The threshold was set as a fold change ≥ 2.0 and a P value < 0.05. GO analysis and KEGG analysis were further applied to determine the roles of these genes with differential expression.

Specifically, RT² Profiler PCR Arrays (Qiagen) in combination with the RT² SYBR Green qPCR Mastermixes and the RT² First Strand Kit were used to analyze the expression of a panel of ion channel genes. The high-quality primer design enable the RT² Profiler PCR Arrays to amplify 96 different gene-specific products simultaneously under uniform cycling conditions. Quantitative RT-PCR reaction was performed on Mastercycler® ep realplex platform (Eppendorf) for 40 cycles: denaturation in 95°C for 15s, annealing in 58°C for 30s, and extension at 68°C for 30s. 2^-ΔΔCT^ method was applied to normalize against housekeeping gene and quantify relative expression. The sequences of primers used are showed in Table 1. Triplicate was done for each sample.

Table1. Primers for real-time qRT-PCR

| Gene | Forward Primer | Reverse Primer |
| --- | --- | --- |
| *Dnmt3a* | CTG GTG ATT GGA GGC AGTCCATGCA | TAGCTGAGGCTGTCTGCATCGGACA |
| *Dnmt1* | GGG CCA GTT GTG TGA CTT GG | CTT GGG CCT GGA TCT TGG GGA |
| *Dnmt3b* | GGATGTTCGAGAATGTTGTGGCC | CAGGTCAGACCTCTCTGGTGACAAG |
| *Kcne1* | TTAGCTACCTCTG CACCGTC | CGCCTCTAGCTTGC TGTCAT |
| *Kcne2* | GGTCTCCTGCATTGCTCACA | CGATGTACTGGTGGTACGGG |
| *Kcne3* | ATTGTCTTCTGGT GCCTCCG | CTGGGCCTATCAGT CCCTCT |
| *Kcnn4* | GTACGGCTGAAA CACCGGAA | GAAAACACAGGAG CAGGGATG |
| *Kcna1* | ATTGGGCGACTTG CTTCTCA | GAGAATTTGACGG GAGGGGG |
| *Kcnq3* | CTGTGCCCACAGC AAAGAAC | GCAATCAGACGTC CTTCCCA |
| *Kcnh8* | CTCACAAGCGTTG GGTTTGG | TCCTCTGCTTGAGT TGCTGG |
| *Kcnj13* | TGCTCCTAGGCCT CATGCTA | ACACGAACGTTGG TCAGAGG |

**Data analysis**

Results were present as mean ± SEM. Data was analyzed using unpaired t-test, one-way ANOVA followed by Tukey’s multiple comparisons test, or two-way ANOVA followed by Sidak’s multiple comparisons test or Bonferroni posttests as appropriate. *P* <0.05 indicates significant difference between groups. Statistical analysis was done with GraphPad Prism 5.0 (GraphPad Software).
